# Primary Malignant Peripheral Nerve Sheath Tumor of the Breast: A Rare Case Report and Review of Literature

**DOI:** 10.7759/cureus.31586

**Published:** 2022-11-16

**Authors:** Muhammad Tahir, Mohamed Zedan, Vindhya Bellamkonda, Donna L Dyess, Eric X Wei

**Affiliations:** 1 Pathology and Laboratory Medicine, University of South Alabama Health Hospital, Mobile, USA; 2 General Surgery, University of South Alabama Health Hospital, Mobile, USA

**Keywords:** sarcoma of breast, metaplastic breast cancer, variant of metaplastic breast cancer, nerve sheet tumor, primary malignant peripheral nerve sheath tumor

## Abstract

Primary breast sarcomas are uncommon and primary mammary malignant peripheral nerve sheet tumors (MPNST) are exceptionally rare. MPNSTs are malignant variants of peripheral nerve sheath tumors. These neoplasms are often associated with neurofibromatosis type I (NF-I) but can also occur sporadically. They tend to occur in the deeper soft tissues, trunk, and extremities.

A 60-year-old Asian female was referred to our surgical clinic for evaluation of a left breast mass and an abnormal mammogram. The patient noticed the mass in the left breast three months earlier and was referred for mammography by her primary physician. Mammography reported partially defined masses in the superior aspect of the left breast, and ultrasound showed a solid mass measuring 5.2 X 3 cm. The mass was 11 cm on clinical exam. Subsequent core biopsy of the left breast lesion showed high-grade malignant neoplasm. Workup showed no evidence of metastatic disease, and the patient underwent modified radical mastectomy. The neoplastic cells were positive for CD99, S-100, SOX-10, neuron specific enolase, p53, vimentin, focally positive for neurofilament, D2-40, p63, and negative for epithelial, melanoma and other sarcoma markers. The tumor was triple negative estrogen receptor (ER), progesterone receptor (PR), and human epidermal growth factor receptor 2 (HER2), with Ki-67 at 61%. A diagnosis of primary high grade malignant peripheral nerve sheath tumor of the breast was rendered. The patient does not have a history of NF-1.

An accurate diagnosis of this rare entity is necessary because it plays a crucial role in the therapeutic options and prognosis. In our case the patient underwent modified radical mastectomy. The purpose of presenting this unique case is to provide awareness of the existence of this entity among pathologists and clinicians for better patient care.

## Introduction

Breast neoplasm is the most common cancer in females worldwide, but malignant breast lesions of mesenchymal lineage are rare [1]. The most common histological variants of primary breast cancer are epithelial in origin comprising invasive ductal adenocarcinoma and lobular carcinoma. Metaplastic carcinomas of breast are not common, and sarcomas are even more rare and account for less than 1% of primary breast cancers [2].

Malignant peripheral nerve sheet tumors (MPNSTs) are an uncommon type of neoplasm and account for 5 to 10% of all neoplastic soft tissue sarcomas. Their incidence is 1:100,000 [3]. MPNSTs are malignant variants of peripheral nerve sheath tumors that originate from large or small peripheral nerves and their tributaries or at the sheaths of peripheral nerve fibers. These neoplasms are counterparts of the benign soft tissue neoplasm like neurofibromas and procure from Schwan cells or pluripotent cells of neural crest origin [4]. These neoplasms are most frequently associated with genetic disorders like neurofibromatosis type I (NF-I) but can also occur sporadically in children and adults [5]. Most commonly these entities occur in the deeper soft tissues, trunk, extremities and usually in the proximity of the nerve trunks. MPNST of the breast is exceedingly rare and has been scarcely reported in the literature. Here we report a sporadic case of MPNST of breast in a 60-year-old female patient with no history of NF-I.

## Case presentation

**Figure 1:**
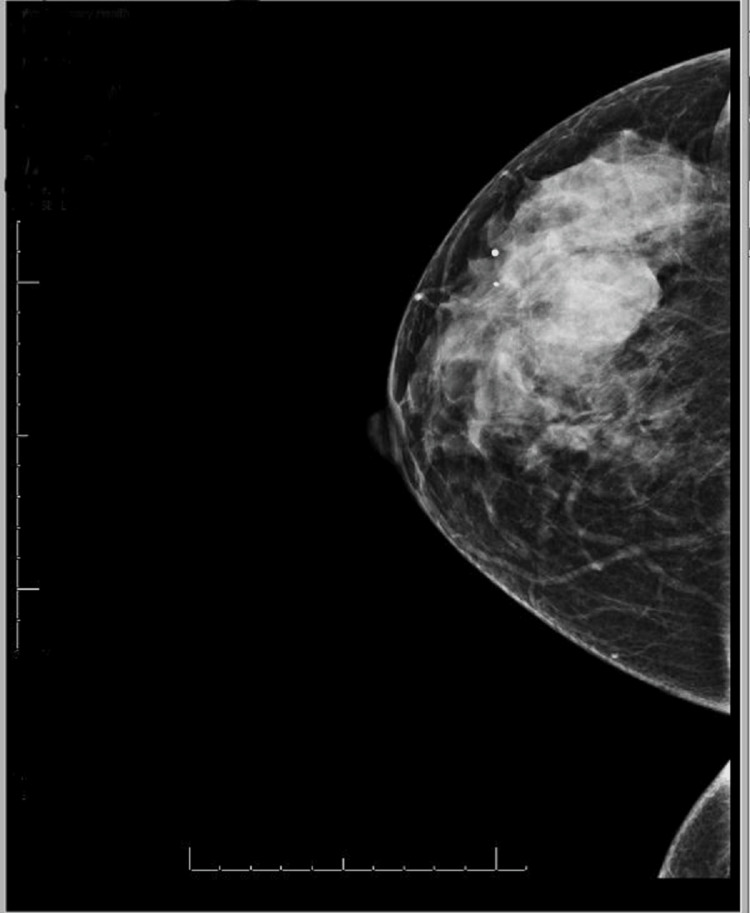
Mammogram showing large, solid homogenous mass.

A 60-year-old Asian female patient was referred to our surgical clinic for evaluation of a left breast mass and an abnormal mammogram. The patient noticed a mass in the left breast three months earlier. Mammography reported partially defined masses in the superior aspect of the left breast, and ultrasound showed a solid mass measuring 5.2 X 3 cm located in the left superior breast (Figure 1).

Subsequent core biopsy of the left breast lesion showed high-grade malignant neoplasm with differential diagnosis of malignant phyllodes tumor, primary sarcoma, and metaplastic carcinoma. There was no evidence of metastatic disease, and the patient underwent a modified radical mastectomy. The excised tumor was well-circumscribed, tan-white, and firm with smooth cut surfaces, measuring 9.6 x 6.1 x 4.3 cm and was located 1.5 cm from the posterior, 5.6 cm from the anterior surgical margin and 5.1 cm from the nipple.

**Figure 2:**
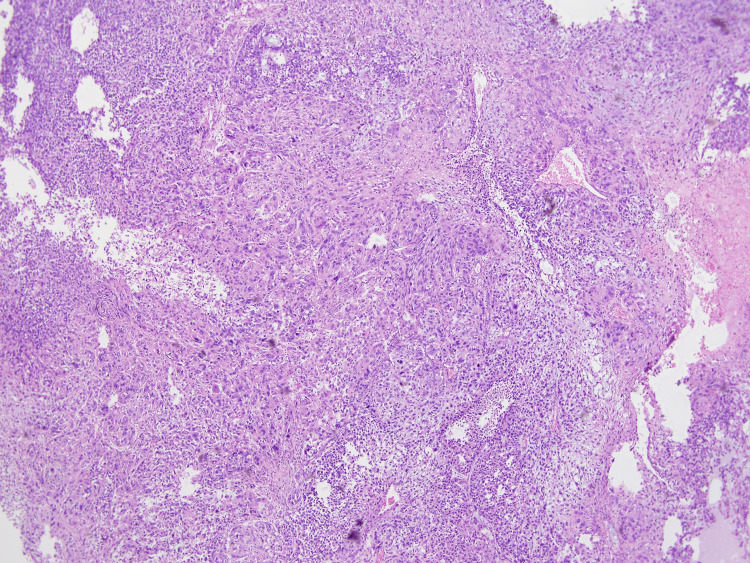
Sheets of spindle to epithelioid neoplastic cells. 4X magnification

Microscopically, the sarcomatous malignant nerve sheath tumor component was predominant, at more than 99% of tumor volume. On low power the tumor was composed of asymmetric spindle to epithelioid cells arranged in dense fascicles with alternating hypocellular and hyper cellular areas with perivascular accentuation (Figure 2).

**Figure 3:**
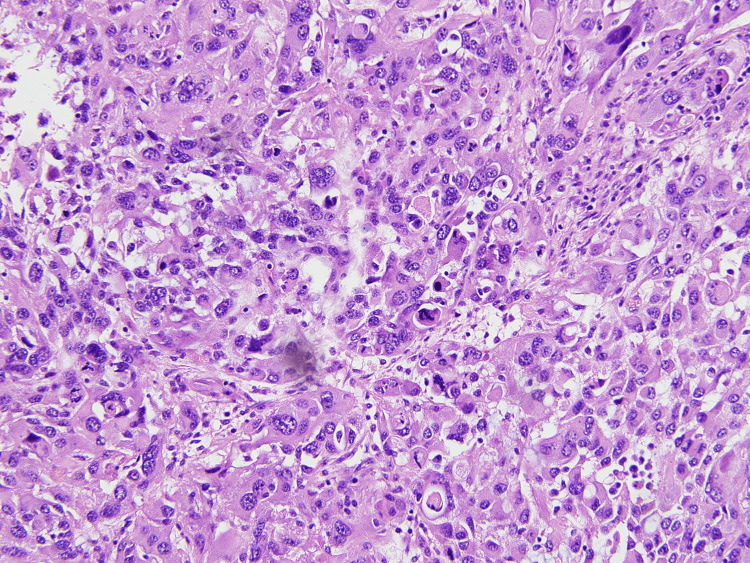
Very pleomorphic, hyperchromatic neoplastic cells with nuclear chromatin clumping and mitosis. 20X magnification

**Figure 4:**
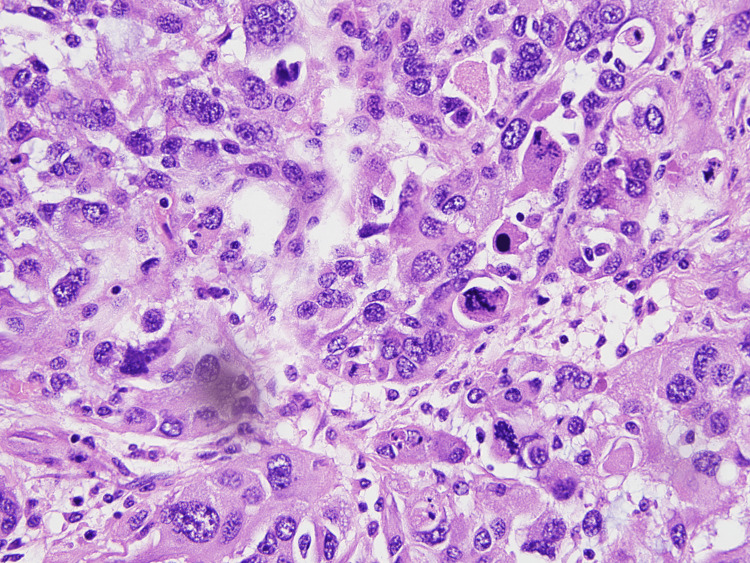
Very pleomorphic, hyperchromatic neoplastic cells with apoptotic bodies and mitosis. 40X magnification

On medium power view very hyperchromatic neoplastic cells with clumped chromatin, think wavy or focally buckled nuclei, with frequent mitosis and apoptotic bodies were evident (Figure 3, 4).

**Figure 5:**
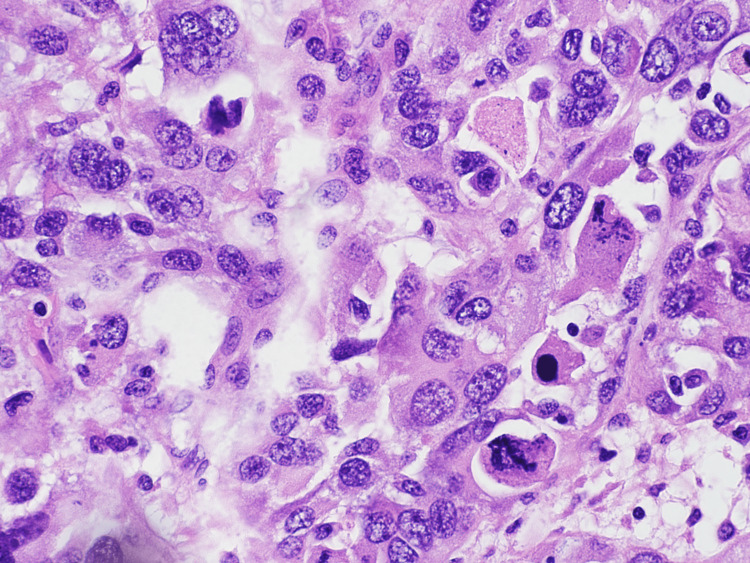
Multinucleated giant cells with atypical mitosis. 60X magnification

Vey pleomorphic, bizarre-looking multinucleated giant tumor cells with atypical mitosis can be seen on high power view (Figure 5). Precursor lesions like neurofibromas and chondrosarcomatous, osteosarcomatous, and rhabdomyosarcomatous heterologous elements were not identified.

**Figure 6:**
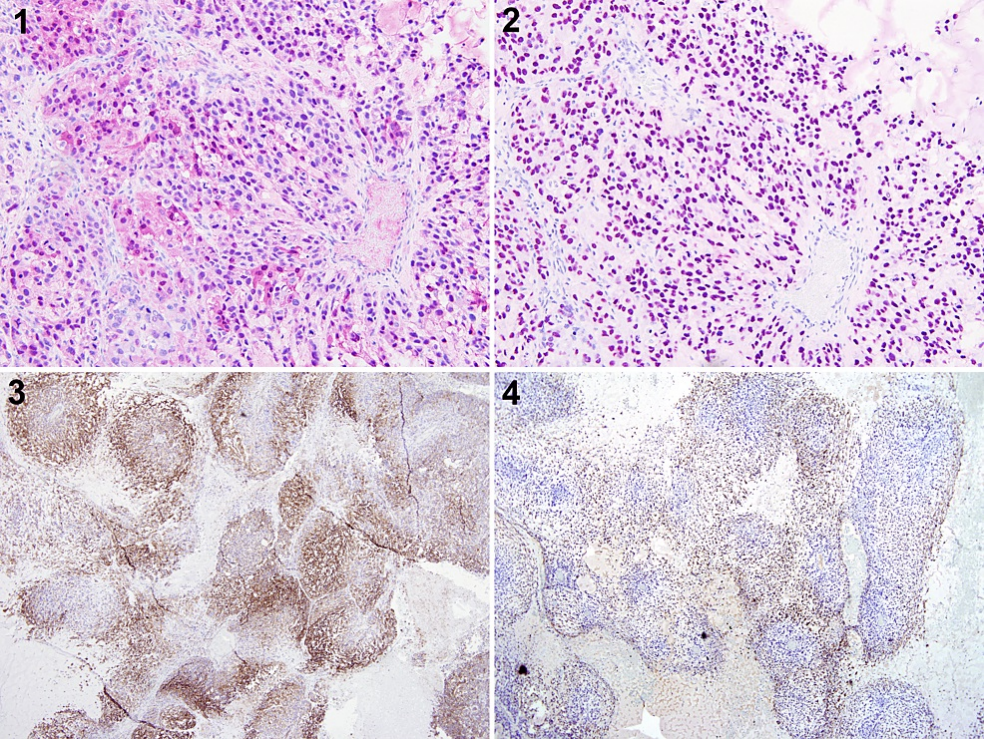
Immunohistochemistry showing positive staining patterns for SOX 10, S100, CD99, and neuron specific enolase (NSE). 4X magnification. 1) SOX 10,  2) S100, 3) CD99, 4) NSE

Immunohistochemically, neoplastic cells were positive for CD99, S-100, SOX-10, neuron-specific enolase, p53, vimentin, and focally positive for neurofilament, D2-40, p63, and negative for epithelial, melanoma, and other sarcoma markers (Figure 6).

The tumor was triple negative estrogen receptor (ER), progesterone receptor (PR), and human epidermal growth factor receptor 2 (HER2), with Ki-67 at 61%. Based on microscopic morphology and immunohistochemistry profile, a diagnosis of primary high-grade malignant peripheral nerve sheath tumor of the breast was rendered. The patient does not have a history of NF-1. There was no lympho-vascular or perineural invasion. After the surgery, the patient was and is being followed up without any complications.

## Discussion

Primary MPNSTs of the breast are exceedingly rare. These malignant variants of peripheral nerve sheath tumors originate from large or small peripheral nerves and their tributaries or at the sheaths of peripheral nerve fibers. These neoplasms are a counterpart of the benign soft tissue neoplasm like neurofibromas and procure from Schwan cells or pluripotent cells of neural crest origin. These neoplasms are often associated with genetic disorders like NF-I but can also occur sporadically in children and adults [3,4].

MPNSTs are categorized into three distinct subtypes: epithelioid, mesenchymal (including Triton tumor), and glandular variants. The epithelioid variant is characterized by its typical histological morphology of rounded spindle cells with plump hyperchromatic nuclei and abundant atypical mitosis as seen in our case [6].

On gross examination, MPNSTs are usually well-circumscribed, round to oval, rarely encapsulated, large, ranging more than 5 cm in diameter, tan-yellow in color, soft to firm in consistency, and often have focal areas of necrosis [7]. The tumor in our case was well-circumscribed, not completely encapsulated, tan-white, firm, and was located 1.5 cm from the posterior margin. Microscopically, the tumor is usually highly cellular and morphologically composed of the spindle to epithelioid cells, with hyperchromatic, thin, wavy, or focally buckled nuclei, abundant mitotic activity with multiple atypical forms in the background of the hyalinized stroma, as seen in our case [8].

Immunohistochemically, the Schwann cells show positive staining for S-100 and SOX-10 CD-57, laminin, and calretinin, and negative for EMA, actin, and various cytokeratins. In contrast, the normal perineural cells are positive for EMA and negative for S-100 protein. The most crucial point that is necessary to highlight is that MPNSTs show some degree of focal expression of S-100 protein instead of diffuse pattern. If a strong and diffuse pattern of S-100 protein expression is identified, the suspected diagnosis would be Schwannoma or metastatic melanoma instead of MPNST [8,9].

In our case the focal and diffuse expression of S-100, SOX-10 showed characteristics of MPNST. Tumor cells were also positive for CD99, neuron-specific enolase (NSE), p53, and vimentin, and negative for CK7, CK5/6, CAM5.2, CD10, CD34, CD117, EMA, E-cadherin, GATA-3, HMB-45, MART-1, myogenin, and muscle specific actin (MSA). The Immunohistochemistry (IHC) results supported and confirmed the diagnosis of MPNST in our case.

In general, MPNSTs behave aggressively and have a high rate of local and distant metastasis. Larger tumor size (greater than 5 cm), presence of NF-1, tumor grade, invasion, and heterologous rhabdomyoma differentiation are the adverse prognostic factors. However, in the literature, no report is available on the median survival or prognosis of MPNSTs of the breast [3,10]. Our patient has not experienced any local reoccurrence or distant metastasis since radical surgical excision. MPNST can affect any gender, age, race, or ethnic group. Multiple cases of sporadic and NF-1-associated MPNST have been reported in the literature. According to our search and availability of online databases, 15 cases have been compiled and listed in Table 1 [10-24].

**Table 1:** Published case reports on malignant peripheral nerve sheath tumors of the breast. SN: Serial Number, S: Sporadic, NF-1: Neurofibromatosis type-1, F: Female, NA: Not Available

SN	Year published	Authors	Age	Gender	Ethnicity	size	Sporadic/Genetic
1	2019	Agarwal R, Sinha D, Tomar R, Mandal S, Bains L, Jain S. Primary malignant peripheral nerve sheath tumor of the breast. Breast J. [10]	32	F	Indian	2.5cm	S
2	2017	Shuayb M, Begum R. Unusual primary breast cancer - malignant peripheral nerve sheath tumor: a case report and review of the literature. J Med Case Rep. [11]	16	F	Indian	11cm	S
3	2018	Bonnet SE, Kang-Chapman JK, Buckley KA, Cui X, Grignol VP, Hawley JR. Malignant peripheral nerve sheath tumor of the breast in a patient with neurofibromatosis 1. Breast J. [12]	36	F	NA	NA	NF-1
4	2007	Dhingra KK, Mandal S, Roy S, Khurana N. Malignant peripheral nerve sheath tumor of the breast: case report. World J Surg Oncol. [13]	38	F	Indian	3.5cm	S
5	2009	Wang H, Ge J, Chen L, Xie P, Chen F, Chen Y. Melanocytic Malignant Peripheral Nerve Sheath Tumor of the Male Breast. Breast Care (Basel). [14]	65	M	Chinese	2cm	S
6	2016	Redzepagic J, Skenderi F, Bajrovic J, Beslagic V, Ibisevic N, Vranic S. Low-grade malignant peripheral nerve sheath tumor: a report of the first case in the breast and literature review. APMIS. [15]	65	F	Bosniaks	2.3cm	S
7	2003	Medina-Franco H, Gamboa-Dominguez A, de La Medina AR. Malignant peripheral nerve sheath tumor of the breast. Breast J. [16]	4	F	Mexican	2.4cm	NF-1
8	2006	Thanapaisal C, Koonmee S, Siritunyaporn S. Malignant peripheral nerve sheath tumor of breast in patient without Von Recklinghausen's neurofibromatosis: a case report. J Med Assoc Thai. [17]	19	F	Thai	NA	NF-1
9	2006	Elsaify W, Elsaify M, Melek R. De novo malignant peripheral nerve sheath tumor of the breast: case report number one. Eur Surg. [18]	18	F	Britch	4cm	S
10	1995	Malas S, Krawitz HE, Sur RK, Uijs RR, Nayler SJ, Levin CV. Von Recklinghausen's disease associated with a primary malignant schwannoma of the breast. J Surg Oncol. [19]	71	F	South Africa	6cm	FN-1
11	2010	Akhator A, Oside CP, Inikori A, Nwanchokor FN. Malignant peripheral nerve sheath tumour: a rare tumour of the breast. Online J Health Allied Sci. [20]	41	F	Nigerian	10cm	NF-1
12	1995	Hauser H, Beham A, Steindorfer P, Schmidt F, Smola MG. Malignant schwannoma of the breast. Langenbecks Arch Chir. [21]	27	F	Australian	1.2cm	S
13	2011	Chalkoo M, Ahangar S, Laharwal A, Patloo A, Mohd A, Dar S. Primary Malignant Peripheral Nerve Sheath Tumor of the Breast—A Case Report. Surg Sci. [22]	60	F	Indian	26cm	S
14	1998	Berrada R, Chahtane A, Lakhdar A, et al. [Malignant schwannoma of the breast. A case report] J Gynecol Obstet Biol Reprod (Paris). [23]	26	F	French	8cm	S
15	2006	Kim HD, Shin HJ, Park YL, Whang KU, Baik SH, Park JS. A case of malignant peripheral nerve sheath tumor arising from neurofibromatosis during pregnancy. Korean J Dermatol. [24]	33	F	Korean	NA	NF-1

## Conclusions

This case report describes a unique case of MPNST of the breast and provides a comprehensive approach to diagnosis, therapeutic options, and prognosis. In our case, the patient was treated with modified radical mastectomy. We suggest proper immuno-histochemical analysis in all the metaplastic breast cancers and sarcomas to find and categorize the unique histological variants that are not yet discovered. An accurate diagnosis of this rare entity is necessary because it plays a crucial role in therapeutic options and prognosis. Long-term follow-up is required and recommended for optimal treatment and better prognosis. The purpose of presenting this unique case is to provide awareness of the existence of this entity to pathologists and clinicians for better patient care. 
